# Adsorption and removal of strontium in aqueous solution by synthetic hydroxyapatite

**DOI:** 10.1007/s10967-015-4228-9

**Published:** 2015-06-20

**Authors:** Yuichi Nishiyama, Tadashi Hanafusa, Jun Yamashita, Yoko Yamamoto, Toshiro Ono

**Affiliations:** Department of Radiation Research, Advanced Science Research Center, Okayama University, 2-5-1 Shikata-cho, Kita-ku, Okayama, 700-8558 Japan; Institute of Plant Science and Resources, Okayama University, 2-20-1 Chuo, Kurashiki, Okayama 710-0046 Japan

**Keywords:** Strontium, Hydroxyapatite, Adsorption, Desorption, Divalent cation

## Abstract

Hydroxyapatite (HAP) is a main mineral constituent of bone and tooth and has an outstanding biocompatibility. HAP is a possible sorbent for heavy metals in wastewater due to its high adsorption capacity and low water solubility. We developed a removal system of ^90^Sr from aqueous solution by HAP column procedure. More than 90 % of ^90^Sr was adsorbed and removed from the ^90^Sr containing solution. Divalent cations, Ca^2+^, had little effect on the removal of ^90^Sr up to a concentration of 1 mmol L^−1^. This clearly indicates that the HAP column technique is advantageous with respect to the capacity to adsorb ^90^Sr from water present in the environment.

## Introduction

Large amounts of radioactive nuclides were released into the environment due to the Fukushima Daiichi Nuclear Power Plant accident on March 11, 2011. The released radioactive nuclides were deposited on the soil, houses, trees, plants, water, and other structures over a wide area of Tohoku as well as the Kanto region [1–4]. Among them, long-lived radioactive nuclides, such as ^134^Cs (half-life: 2.06 year), ^137^Cs (half-life: 30.17 year), and ^90^Sr (half-life: 28.79 year), which are considered to be harmful to humans, are of great concern in terms of environmental contamination. Environmental monitoring has been performed by detecting γ-rays [5]. The long-lived γ-emitter ^137^Cs has been used to evaluate decontamination, because it is easily detected using various instruments including GM and scintillation counters, and is more precisely determined with a Ge semiconductor detector. However, the pure β^−^-emitter ^90^Sr requires a complicated extraction and purification process for analysis. Therefore, few studies on ^90^Sr release have been performed so far.

There are several methods to remove of metal ions from wastewater, such as chemical precipitation [6], ion exchange [7], membrane treatment [8], and adsorption [9, 10]. Adsorption is one of the most commonly used methods due to its simplicity and selectivity. For the separation of strontium ions, various types of organic [11, 12] and inorganic [13] adsorbents have been reported. Hydroxyapatite (HAP, Ca_10_(PO_4_)_6_(OH)_2_) is the main mineral constituent of bones and teeth. It has been widely used as a biomaterial for hard tissues because of its marked biocompatibility [14]. It possesses a high affinity for proteins and has been applied for separating them [15]. Recently, HAP has attracted particular interest in treating wastewater containing heavy metals due to its high adsorption capacity and low water solubility [16–19].

The aim of this study was to develop a system to remove strontium from aqueous solution with an adsorption procedure. We focused on HAP prepared by a different calcination temperature as sorbent materials, and showed the effective adsorption and desorption of strontium with the HAP column procedure.

## Experimental

### Reagent

Strontium nitrate was obtained from Wako Pure Chemical Industries (Osaka, Japan). Sr standard solution (1 mg mL^−1^) for atomic absorption spectrometry was also from Wako Pure Chemical Industries. Ultrapure HNO_3_ was purchased from Sigma Aldrich (Tokyo, Japan). ^90^SrCl_2_ (1.05 × 10^4^ Bq mL^−1^) in 0.1 N HCl was purchased from Japan Radiation Association (Tokyo, Japan).

### Sorbents

**Fig. 1:**
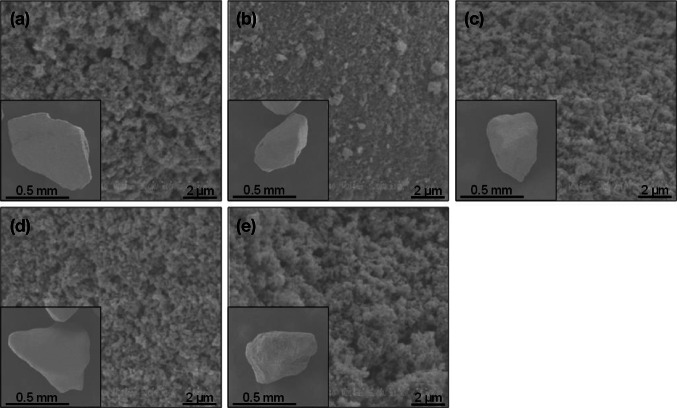
SEM images of HAP particles. **a** HAP-1, **b** HAP-2, **c** HAP-3, **d** HAP-4, and **e** HAP-5

Several kinds of synthetic HAP and tricalcium phosphate (TCP) were prepared (Taihei Chemical Industrial, Co., Ltd., Osaka, Japan). Their characteristics (calcination temperature and particle size) are listed in Table 1. The morphology of HAP particles was examined using scanning electron microscopy (S-3200 N, Hitachi, Ltd., Tokyo, Japan) and is illustrated in Fig. 1. Synthetic zeolite (Type A3) was purchased from Wako Pure Chemical Industries.

**Table 1:** Physico-chemical characteristics of sorbents

Sorbents	Calcination temperature (°C)	Particle size (mm)
Hydroxyapatite		
HAP-1^a^	200	ND
HAP-2	500	0.25–0.35
HAP-3	900	0.25–0.35
HAP-4	900	0.35–0.50
HAP-5^a^	900	ND
Tricalcium phosphate		
TCP-1	200	0.15–0.25
TCP-2	200	0.25–0.35
TCP-3	200	0.35–0.50
TCP-4	200	0.50–0.84
TCP-5	500	0.25–0.35
TCP-6	900	0.25–0.30
TCP-7	900	0.35–0.50
TCP-8	1250	0.35–0.50
TCP-9	1250	1.7–2.8
Zeolite		
Zeolite A3	–	0.50–1.18

^a^Their sizes are not different from HAP-2, HAP-3, and HAP-4, as shown in Fig. 1

*ND* not determined

### Batch sorption studies

Adsorption studies were carried out using the batch technique. One milliliter of Sr^2+^ solution (100 μg L^−1^) was mixed with 0.02–0.12 g of HAP, TCP, or zeolite particles, respectively. The initial pH was adjusted to values ranging from 4.0 to 9.6 using 1 mmol L^−1^ sodium acetate buffer, phosphate buffer, and carbonate buffer, respectively. Each mixture was incubated for 10 or 60 min at room temperature with shaking. After incubation, they were centrifuged at 6000 rpm for 5 min and the supernatant was collected. The Sr^2+^ concentration in the supernatant was determined by atomic absorption spectrometry. The adsorption efficiency (*A*_eff_) was calculated as:

1 $$ A_{\text{eff}} (\% ) = 100 \times \left( {1 - \frac{C}{{C_{0} }}} \right) $$

where *C*_0_ is the initial Sr^2+^ concentration (μg L^−1^) in the solution and *C* is the Sr^2+^ concentration (μg L^−1^) in the supernatant.

### Adsorption equilibrium

Adsorption equilibrium studies were performed by equilibrating 0.1 g of HAP with 10 mL of Sr^2+^ solution at varying concentrations (0–1000 mg L^−1^) at 20 °C and at an initial pH of 7.0. After 24-h incubation, the reaction mixtures were centrifuged at 6000 rpm for 5 min and aliquots of supernatants were determined for the Sr^2+^ concentration by atomic absorption spectrometry. The amount of Sr^2+^ adsorbed at equilibrium, *q*_e_ (mmol g^−1^) was calculated as:

2 $$ q_{e} = \frac{{C_{0} - C_{e} }}{{C_{s} }} $$

where *C*_0_ is the initial Sr^2+^ concentration (mmol L^−1^), *C*_e_ is the residual Sr^2+^ concentration at equilibrium (mmol L^−1^), and *C*_s_ is the HAP concentration (g L^−1^).

### Adsorption of ^90^Sr

A tracer amount of ^90^Sr^2+^ was added to 100 μg L^−1^ of Sr(NO_3_)_2_ solution, containing divalent cations (Ca^2+^ and Mg^2+^) in the range of 0–100 mmol L^−1^. Adsorption of 1 mL ^90^Sr solution (105 Bq mL^−1^, pH 7.0) was evaluated using column method. Radioactivity of the supernatants or eluates as the total beta activity was measured by a low background gas flow beta counter (LBC-4202, Hitachi Aloka Medical, Ltd., Tokyo, Japan).

### Condensation and separation using column apparatus

Sr^2+^ was adsorbed and condensed on an HAP-filled column. A total of 0.2 g HAP was emerged in 1 mL of distilled water and transferred to a 0.8 × 4-cm chromatography column (Bio-Rad Laboratories, Inc., Hercules, CA, USA). The column was washed and equilibrated twice with phosphate buffer (pH 7.0). Then, 10 mL of Sr^2+^ solution (15 ng mL^−1^, pH 7.0) was loaded and the effluent was collected. After the adsorption of Sr^2+^ onto the HAP column, it was eluted by adding a total of 5 mL of 100 mmol L^−1^ Ca^2+^ ion solution, and 1 mL of each eluate fraction was collected. The Sr^2+^ concentration of each eluate was measured by atomic absorption spectrometry.

### Atomic absorption spectrometry

A flame atomic absorption spectrometer (Z-9000, Hitachi, Ltd., Tokyo, Japan) equipped with a hollow cathode lamp (wave length, 490 nm) was used for Sr^2+^ determination. Analytical working solutions containing 0, 10, 25, 50, and 100 μg L^−1^ of Sr^2+^ were prepared by the appropriate dilution of 1 g L^−1^ standard solution with ultrapure nitric acid. The absorbance of blank, analytical solution, and sample solution was measured successively with the optimized operating conditions.

## Results

### Batch adsorption

**Fig. 2:**
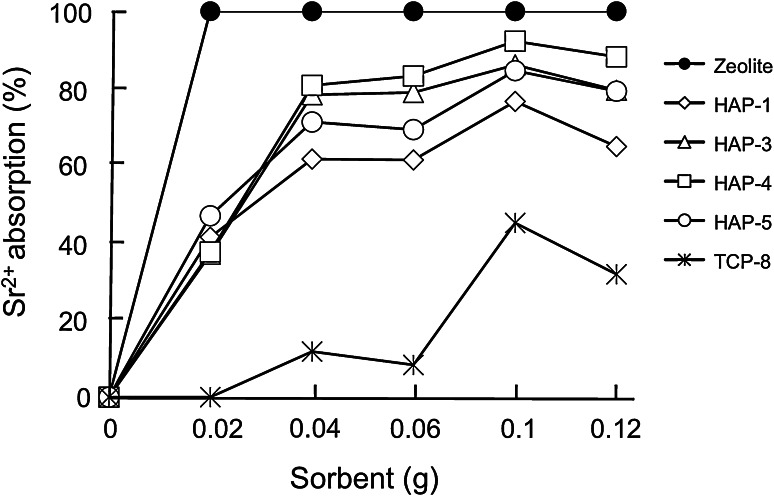
Effect of sorbent amount on Sr^2+^ adsorption

**Fig. 3:**
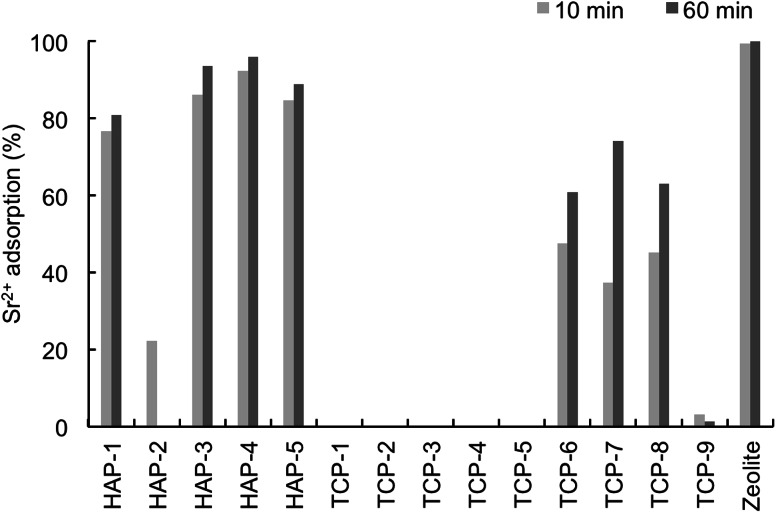
Sr^2+^ adsorption efficiency of HAP, TCP, and zeolite. Analysis by batch method

Five kinds of HAP particle and nine kinds of TCP particle were tested for their Sr^2+^ adsorption properties with the batch method. The effect of the amount of sorbent was investigated. As shown in Fig. 2, the adsorption of Sr^2+^ rose with an increasing amount of HAP, and reached a maximum plateau at 0.04 g of HAP. A total of 0.05 g of each sorbent and 1 mL of Sr^2+^ solution were thus incubated for 10 and 60 min. Four kinds of HAP particle, HAP-1, HAP-3, HAP-4, and HAP-5, adsorbed Sr^2+^ as efficiently as zeolite particles (Fig. 3). However, TCP particles showed less adsorption efficiency. We, therefore, choose HAP-1, HAP-3, HAP-4, and HAP-5 particles for further studies.

### Effect of pH

**Fig. 4:**
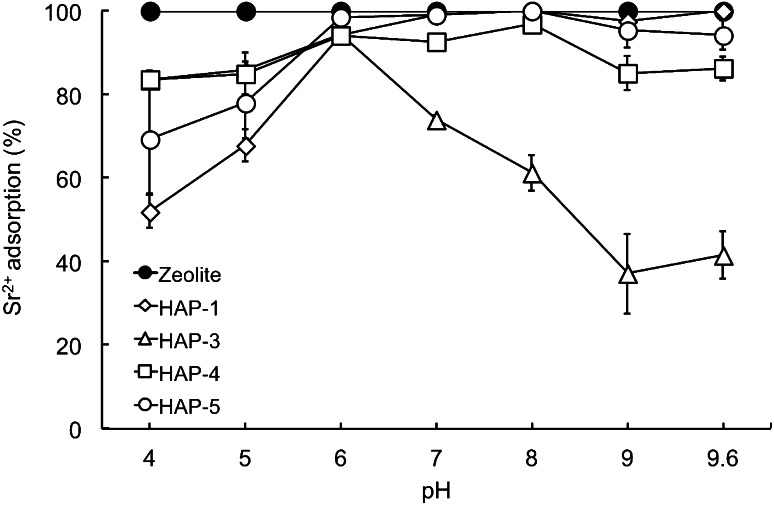
Effect of pH on Sr^2+^ adsorption by HAP. Each value represents the mean ± SD of three independent experiments

The effect of the pH on Sr^2+^ adsorption by the selected HAP particles was investigated. As shown in Fig. 4, the adsorption of Sr^2+^ increased with pH elevation. The plateau adsorption value reached pH 6, and was maintained in an alkaline pH range except for with HAP-3 particles.

### Adsorption isotherm

**Fig. 5:**
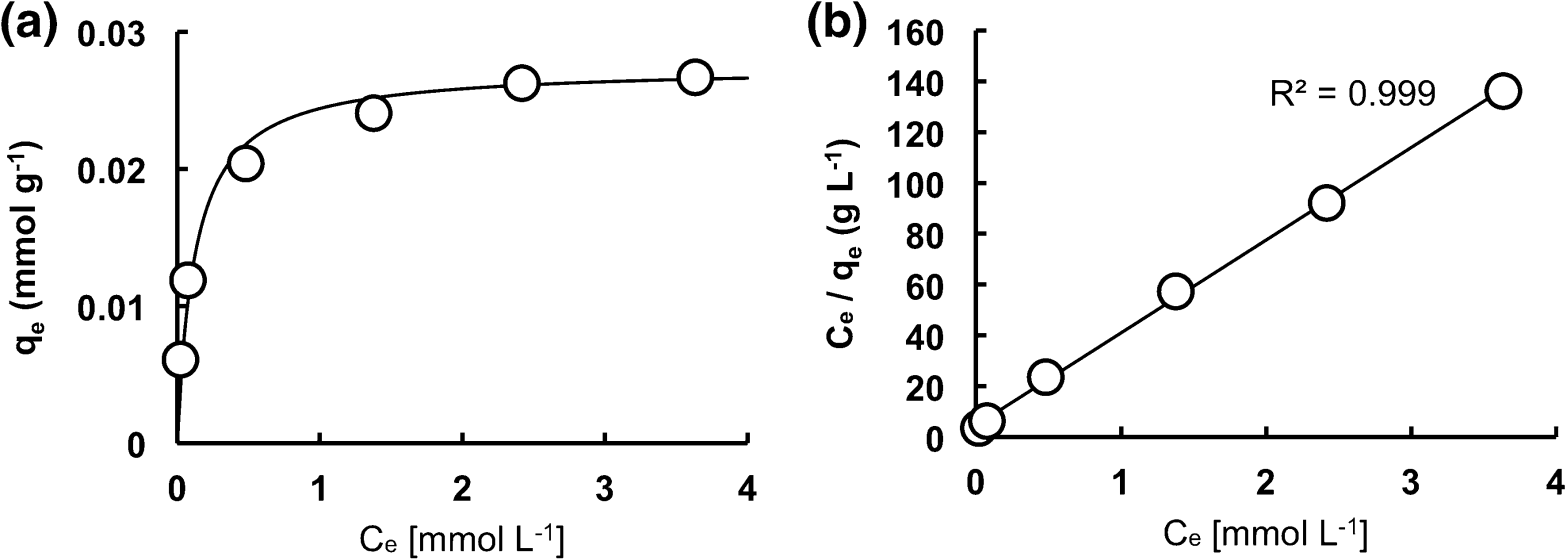
Equilibrium adsorption of Sr^2+^ on HAP. **a** Adsorption isotherm. **b** Langmuir isotherm plot

Figure 5a shows the adsorption isotherm of HAP-5 particles at 20 °C. The adsorbed amount of Sr^2+^ rapidly increased and then gradually reached a maximum plateau. The adsorption curve corresponded to the Langmuir isotherm. The Langmuir isotherm was applied for equilibrium adsorption using the following equation:

3 $$ \frac{{C_{e} }}{{q_{e} }} = \frac{1}{{q_{\hbox{max} } \cdot K}} + \frac{{C_{e} }}{{q_{\hbox{max} } }} $$

where *C*_e_ (mmol L^−1^) is the equilibrium concentration of Sr^2+^, *q*_max_ (mmol L^−1^) is the maximum amount of Sr^2+^ adsorbed on the HAP at equilibrium, and *K* (L mmol^−1^) is a constant related to the adsorption. The constants of *q*_max_ and *K* were evaluated by the slope and intercept of the plot drawn between *C*_e_*/q*_e_ versus *C*_e_ (Fig. 5b). The *q*_max_ and *K* value were determined to be 27 μmol g^−1^ and 8.0 L mmol^−1^, respectively.

### Column adsorption and separation

**Fig. 6:**
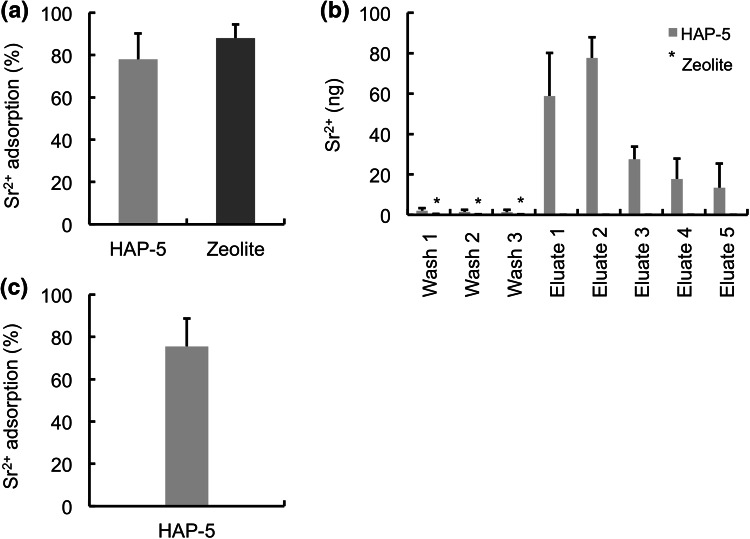
Column method. **a** Sr^2+^ adsorption onto HAP and zeolite column. **b** Desorption of Sr^2+^ by adding 0.1 mol L^−1^ CaCl_2_ solution as the eluent. **c** Reuse of HAP column. After stripping Sr^2+^ from the column, Sr^2+^ solution was re-applied onto the column. Each value represents the mean ± SD of three independent experiments

In order to investigate the column adsorption efficiency, 10 mL of Sr^2+^ solution (15 ng mL^−1^, pH 7.0) was applied onto the HAP-5 and zeolite-loaded columns equilibrated with 1 mM phosphate buffer (pH 7.0), respectively. As shown in Fig. 6a, nearly 80 and 90 % of Sr^2+^ were adsorbed on the HAP-5 and zeolite columns, respectively. We conducted stripping of Sr^2+^ from the loaded columns using 100 mmol L^−1^ CaCl_2_ solution. A 1-mL fraction was collected at each step for Sr^2+^ determination using a flame atomic absorption spectrometer. Sr^2+^ was completely eluted from the HAP-5 column in the first to fifth fractions (Fig. 6b). However, no Sr^2+^ was stripped from the zeolite column. Ten milliliters of Sr^2+^ solution (15 ng mL^−1^, pH 7.0) was then re-applied onto the Sr^2+^-stripped HAP-5 column. As shown in Fig. 6c, Sr^2+^ was successfully adsorbed onto the regenerated HAP-5 column.

## ^90^Sr adsorption

**Fig. 7:**
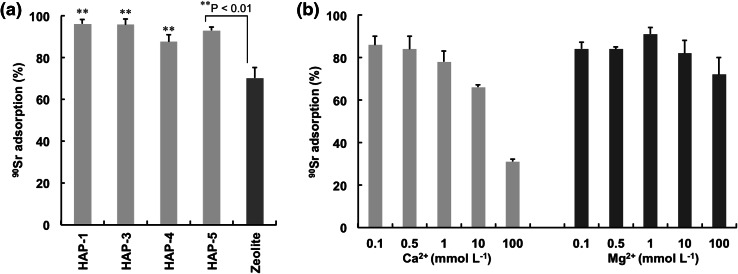
Adsorption of ^90^Sr onto HAP and zeolite column. **a** Adsorption efficiency from competing ion free solution. **b** Effect of competing Ca^2+^ and Mg^2+^ ions analyzed by HAP-5 column. Each value represents the mean ± SD of three independent experiments. *P < 0.01 by unpaired Student’s *t* test

We performed ^90^Sr adsorption tests with column operation technique. The effect of competing divalent cations (Ca^2+^ and Mg^2+^) on the adsorption was evaluated as a function of cation concentrations using the HAP-5 column. As shown in Fig. 7a, more than 90 % of ^90^Sr was adsorbed onto all HAP particles examined from competing cation-free solution. On the other hand, zeolite was less effective regarding its ^90^Sr adsorption performance compared with HAP particles. Adsorption of ^90^Sr was not influenced by the Ca^2+^ concentration up to 1 mmol L^−1^. It decreased to 30 % in the presence of 100 mmol L^−1^ Ca^2+^. Mg^2+^ had little effect on the removal of ^90^Sr over the entire concentration range (Fig. 7b).

## Discussion

In this study, we developed an effective method for separating strontium from aqueous solution on HAP particles with a column procedure. Nearly 80 % of Sr^2+^ was adsorbed on the HAP column. The adsorption equilibrium fitted the Langmuir isotherm equation. The maximum adsorption capacity was 27 μmol g^−1^. The adsorbed Sr^2+^ was successfully stripped from the column using a small amount of Ca^2+^ solution. It resulted in 30–50 % condensation of Sr^2+^ from that in the initial solution. The regenerated column could be used for the second round of separation.

Zeolite has been traditionally used to remove radionuclides including ^137^Cs and ^90^Sr from radioactive wastewater, by ion exchange with Na^+^ ions it contains [10, 20]. Zeolite showed almost the complete adsorption and removal of ^90^Sr with the batch operation technique in our study. Using the column operation technique, however, it showed less effective ^90^Sr adsorption compared to that of HAP. Furthermore, different from the HAP column, desorption of Sr^2+^ from the zeolite column was not achieved. It clearly indicated that the HAP column operation technique had advantages with respect to its desorption capacity, environmental safety, and disposal of the unloaded column compared with the zeolite column.

The adsorption mechanism of HAP depends on several factors, such as the solution pH [21], physico-chemical properties [22], and presence of other metal ions [23]. Metal ion adsorption onto HAP is pH-dependent. The lower adsorption in the acidic solutions is attributed to the H^+^ ions competing with the metal ions for exchange sites. We showed that Sr^2+^ adsorption increased with pH elevation. The highest adsorption was observed at a pH above 6.0.

The crystallinity of HAP has been shown to correlate with the metal ion adsorption behavior. Stötzel et al. [24] and Rosskopfová et al. [19] showed that highly crystalline HAP particles, which were calcined at elevated temperatures of 700–1000 °C, had a lower adsorption capacity than low-level crystalline HAP particles. In this study, however, HAP particles calcined at 200 and 900 °C exhibited the same adsorption performance, indicating the absence of a correlation between crystallinity and Sr^2+^ adsorption.

Tricalcium phosphate (Ca_3_(PO_4_)_2_, TCP) exhibits Sr^2+^ adsorption with a lower activity than HAP [25]. TCP does not show an apatite structure and the Sr^2+^ is thought to enter the heterogeneous structure of TCP with chemical reactions. We tested 9 kinds of TCP with different particle sizes, which were prepared by calcination at different temperatures (200–1250 °C). TCPs calcined at higher temperatures showed a moderate Sr^2+^ adsorption behavior. However, no Sr^2+^ adsorption was observed on TCPs calcined at a lower temperature (200 °C).

Another factor influencing Sr^2+^ adsorption is the presence of competing cations. Sr is an alkaline-earth metal element and it behaves similarly to Ca, Mg, and Ba. Smičiklas et al. [26] reported the effect of alkaline-earth metal ions (Ca^2+^ and Mg^2+^) and alkaline metal ions (Na^+^ and K^+^) on Sr^2+^ adsorption onto HAP using the batch method. Competing cations decreased by 60–70 % on Sr^2+^ adsorption. Natural samples contain large quantities of Na^+^, K^+^, and Ca^2+^. The separation of tracer amounts of ^90^Sr from these cations is particularly important. In our study, Mg^2+^ had little effect on the removal of ^90^Sr. The adsorption of ^90^Sr was not influenced in the presence of 1 mmol L^−1^ Ca^2+^. It decreased by 20 % with an increase of Ca^2+^ to 10 mmol L^−1^. The average amount of Ca^2+^ in natural water is 15–20 mg L^−1^ (0.375–0.5 mmol L^−1^) in Japan [27]. In some areas of Europe, the Ca^2+^ level in natural water is several times higher than that in Japan, but does not exceed 10 mmol L^−1^ [28]. Taken together, the present HAP column technique is useful to remove ^90^Sr from the wastewater as well as natural water in the environment.

## Conclusion

In this study, we developed a system to remove strontium from aqueous solution with an HAP column procedure. Divalent competing cations, Ca^2+^, had little effect on the removal of ^90^Sr. Our system may be useful to remove ^90^Sr from wastewater.
